# Author Correction: All-optical switching based on plasmon-induced Enhancement of Index of Refraction

**DOI:** 10.1038/s41467-022-35148-x

**Published:** 2022-12-12

**Authors:** Rakesh Dhama, Ali Panahpour, Tuomas Pihlava, Dipa Ghindani, Humeyra Caglayan

**Affiliations:** Tampere University, Faculty of Engineering and Natural Sciences, 33720 Tampere, Finland

**Keywords:** Metamaterials, Nanophotonics and plasmonics

Correction to: *Nature Communications* 10.1038/s41467-022-30750-5, published online 03 June 2022

The original version of this article missed discussion and citation of some related works. This has now been added as new reference 16 to the HTML and PDF version of the article.

The fourth sentence of the first paragraph of the introduction incorrectly read as “Other switching schemes involve the hybridization of metamaterials by functional components such as semiconductors, carbon nanotubes, graphene, liquid crystals, and phase-change materials^10–15^.”

This is now corrected as “Other switching schemes involve the hybridization of metamaterials by functional components such as semiconductors, carbon nanotubes, graphene, liquid crystals, and phase-change materials^10–15^ and a metamaterial of plasmonic circuits^16^.”

The first sentence of the second paragraph of the Introduction missed a citation as “Linear interference phenomena are introduced as a promising approach to achieving ultrafast all-optical switching^16,17^ requiring arbitrarily low-intensity optical beams down to the level of single-photon regime^18^.”

This is now corrected as “Linear interference phenomena are introduced as a promising approach to achieving ultrafast all-optical switching^16–18^ requiring arbitrarily low-intensity optical beams down to the level of single-photon regime^19^.”

Section Results and Discussion, subsection Experimental Results and Simulations, eighth paragraph, first sentence incorrectly read as “The extraordinary enhancement in output signal intensity (*x*-polarized light) is attributed to electromagnetically induced negative absorption in the horizontal nanoantenna via coherent control of surface plasmon resonances.”

This is now revised as “The extraordinary enhancement in output signal intensity (*x*-polarized light) is attributed to electromagnetically induced negative absorption in the horizontal nanoantenna via coherent control of surface plasmon resonances and this mechanism is different compared to previous work^16^ which is based on the interference and depends on the angle of incidence, and the modulation is only observed for non-normal incidence.”

This has been corrected in both the PDF and HTML versions of the Article.

**Reference**

[16] Davis, T. J., Gomez, D. E. & Eftekhari, F. All-optical modulation and switching by a metamaterial of plasmonic circuits. *Opt. Lett.* **39**, 4938–4941 (2014).

