# Supplementary material for: All-optical switching based on plasmon-induced Enhancement of Index of Refraction
Source: Nat Commun. 2022 Jun 3;13:3114. doi: 10.1038/s41467-022-30750-5 (PMC9166808; doi:10.1038/s41467-022-30750-5)
Supplement: Supplementary file 1 — Supplementary Information [file 41467_2022_30750_MOESM1_ESM.pdf]

Supplementary Information: All-optical switching based on plasmon-induced Enhancement of Index of Refraction

RAKESH DHAMA,[1], ALI PANAHPOUR,[1] TUOMAS PIHLAVA,[1] DIPA GHINDANI,[1] AND HUMEYRA CAGLAYAN,[1*]

[1] *Faculty of Engineering and Natural Sciences, Photonics, Tampere University, 33720 Tampere, Finland*
[*] *humeyra.caglayan@tuni.fi*

Supplementary Note 1: Spectral Response of the Control Beam

In Figure 3 of the manuscript, signal beam transmittance minima is observed at 800 nm with phase difference of $\delta\phi = \pi/2$. While with the opposite phase difference of $\delta\phi = -\pi/2$, the remarkable enhancement in signal transmission appears at the cost of control transmission. Supplementary Figure 1 shows decrease in the transmission of control beam by means of numerical simulations (Supplementary Figure 1 (a)) and experiments (Supplementary Figure 1 (b)). This clearly confirms energy converting from one polarization (control beam) to another (signal beam) due to the mutual coherence condition between the beams. Note, that the relative enhancement in signal transmission is much higher than the decrease in control transmission, due to the input intensity of control being significantly higher than signal input. Thus, a small decrease in control intensity is enough to give rise to high signal transmittance enhancement. As shown in Supplementary Figure 1 (a), 13% decrease in control beam intensity can enable up to 16×13% = 208% amplification in output signal intensity for $C_{\text{amp}} = 4$. This is in complete agreement with the simulated 206% enhancement of output signal intensity (see Figure 3(c) in the manuscript).

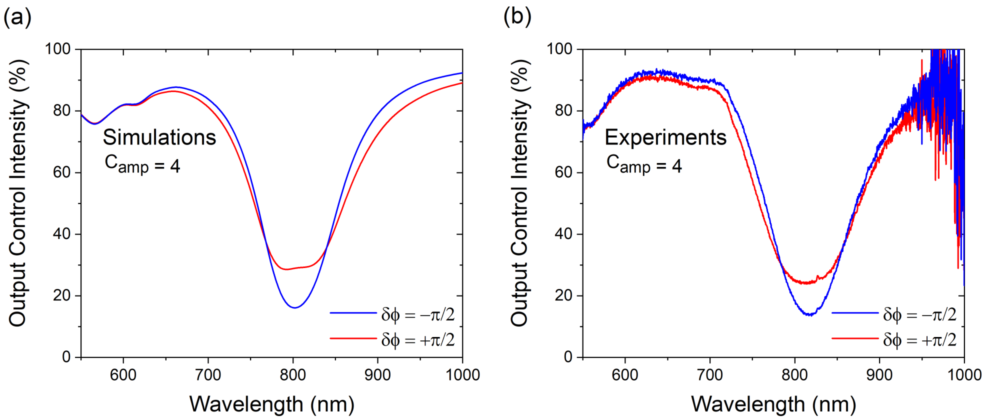

Supplementary Figure 1. (a) Numerically simulated and (b) measured output intensity curves of control beam normalized to control input intensity for $C_{\text{amp}} = 4$ as the function of phase differences of $\delta\phi = \pm\pi/2$ between control and signal beam.

Supplementary Figure 2 reports the broad transparency in terms of full width at half maximum (FWHM) of output signal intensity curves as the function of Camp values $C_{\text{amp}} = 3, 4$ in comparison to the intrinsic response of the metasurface centered at 800 nm and confirms broadband transparency almost in the whole plasmon band of the metasurface with FWHM of 100 nm.

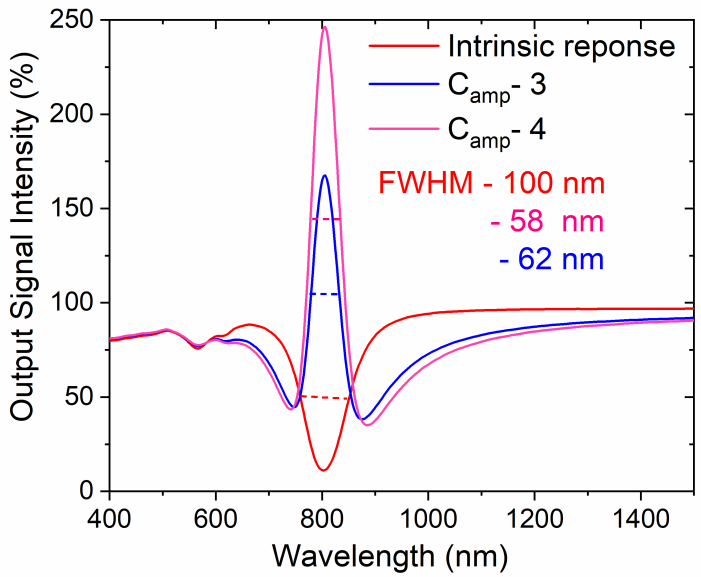

Supplementary Figure 2. Broadband transmission enhancement of signal beam in plasmon band of the metasurface as a function of amplitudes of control beam ($C_{\text{amp}} = 3, 4$) with phase difference of $\delta\phi = \pm\pi/2$ in comparison to the intrinsic response of the metasurface when there is no polarizer and phase delay component included in optical system.

Supplementary Note 2: Modulation in Signal Beam with different phase values

The intensity of the transmitted signal depends on the control intensity and the phase difference between the signal and control beams. However, phase shifts that result in higher modulation strength are more desirable. We have performed some analytical calculations and numerical simulations for different phase shifts when $C_{\text{amp}} = 4$. Supplementary Figure 3 and 4 show that phase shifts $\pm\pi/2$ and $0 - \pi$ can result in higher modulation strengths (but at slightly different wavelengths) compared to other phase shift values such as $\delta\phi = \pm\pi/3$ and $\delta\phi = \pm\pi/4$.

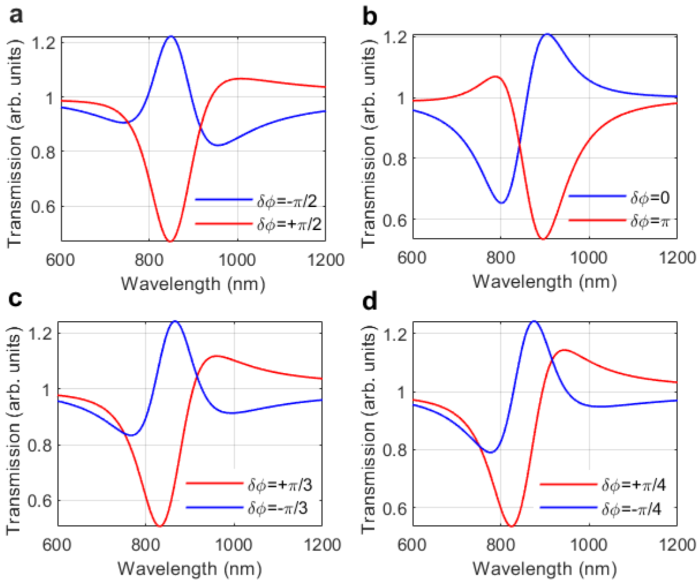

Supplementary Figure 3. Analytically calculated signal transmission when $C_{\text{amp}} =$ 4 and the phase difference between signal and control is (a) $\delta\phi = \pm\pi/2$, (b) $\delta\phi = \pi$, $\delta\phi = 0$, (c)$\delta\phi = \pm\pi/3$ and (d) $\delta\phi = \pm\pi/4$.

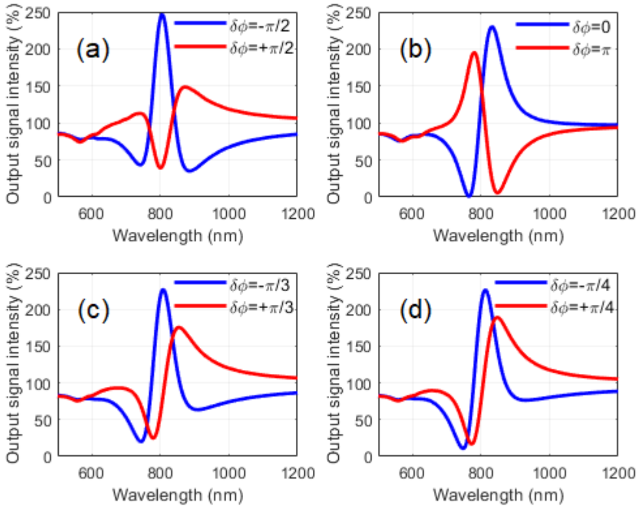

Supplementary Figure 4. Numerically simulated output signal intensity when C_{amp} = 4 and the phase difference between signal and control is (a) $\delta\phi = \pm\pi/2$, (b) $\delta\phi = \pi$, $\delta\phi = 0$, (c)$\delta\phi = \pm\pi/3$ and (d) $\delta\phi = \pm\pi/4$.

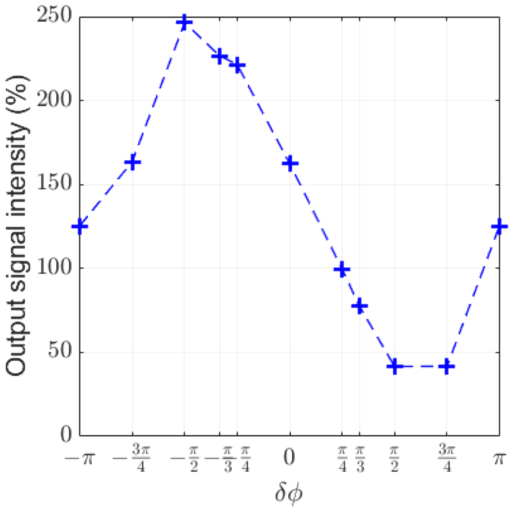

Supplementary Figure 5. Signal transmission for $C_{amp} = 4$ at 806 nm (the resonance peak wavelength of $\delta\phi$ = -$\pi/2$) for different values of $\delta\phi$ acquired via FDTD simulations.

Supplementary Figure 5 visualizes how changing the phase shift affects output signal intensity at 806 nm where the transmission peak is located for $\delta\phi = -\pi/2$.
